# Case Report: Metabolic, inflammatory, and neurological improvements after a ketogenic diet in a woman with treatment-resistant schizophrenia and metabolic syndrome

**DOI:** 10.3389/fpsyt.2025.1710785

**Published:** 2025-11-20

**Authors:** Sidney L. Murray, Gopal Vyas, Erich Eberhardt, Daniel J. O. Roche, Heather A. Adams, Valerie Harrington, AnnMarie Kearns, Matthew Glassman, Alexa Yuen, Joshua Chiappelli, Sharon Pugh, Bobbie Barron, Kay Sandow, Christopher M. Palmer, Deanna L. Kelly

**Affiliations:** 1 Maryland Psychiatric Research Center, University of Maryland School of Medicine, Catonsville, MD, United States; 2 Spring Grove Hospital Center, Maryland Department of Health, Catonsville, MD, United States; 3 Institute for Clinical and Translational Research, Johns Hopkins University School of Medicine, Baltimore, MD, United States; 4 Department of Psychiatry, Harvard Medical School, Boston, MA, United States

**Keywords:** ketogenic diet, schizophrenia, metabolic syndrome, inflammation, insulin-resistance, extrapyramidal side effects

## Abstract

We report the case of a 48-year-old woman with treatment-resistant schizophrenia (TRS) and metabolic syndrome who completed a 5-week medical ketogenic diet (KD) in an inpatient setting. The patient was initially started on a 2.5:1 ratio of fat to combined carbohydrate and protein in grams, but her diet was modified on two occasions due to difficulty reaching consistent ketosis (βHB ≥0.5 mmol/L). Ketosis became more consistent throughout the study but was only fully maintained the last 5 days. Despite this, the patient had many clinically meaningful improvements including a 69% decrease in insulin-resistance (HOMA-IR), 41% decrease in C-peptide, and a 64% decrease in fasting insulin, despite minimal weight loss. Importantly, insulin-resistance moved from pre-diabetic to optimal levels. Fingerstick glucose also decreased. CRP reduced by 61%, suggesting movement from high to average cardiac risk. Extrapyramidal side effects (i.e., pseudoparkinsonism) improved dramatically (80% decrease), reaching almost full resolution. Global psychopathology ratings were not improved; however, the participant had only a few days consistently in ketosis and was facing significant personal stressors nearing the endpoint, which may have eclipsed clinical benefits. Despite this, we saw a hint of improvement in negative symptoms, which we point out as these are particularly problematic in TRS. These results are congruent with emerging data suggesting various health benefits of KD for people with schizophrenia, and we report for the first time its impact in TRS with long-term use of antipsychotic medications (clozapine, olanzapine) that contribute to metabolic syndrome, parkinsonian-like symptoms, and cardiac risk. Our results suggest that despite TRS, dual antipsychotic treatment and limited time in ketosis, a KD can reverse insulin resistance, greatly improve antipsychotic-associated pseudoparkinsonism, and reduce cardiac risk and inflammation. Thus, this diet may be a beneficial treatment alongside antipsychotic medication. We also suggest that well-controlled clinical trials longer than 5 weeks and with consistent ketosis are needed. Additionally, lower calories or a higher ratio of fat to combined protein and carbohydrate may be necessary to maintain ketosis for individuals with metabolic dysfunction who are taking antipsychotic medication.

## Background

Schizophrenia (SZ) is a serious mental illness with positive symptoms (e.g., delusions, hallucinations), negative symptoms (e.g., alogia, blunted affect), and cognitive symptoms (e.g., poor attention, slow processing speed) (1). SZ affects 1% of the population (2, 3), and 5% of individuals with SZ die by suicide (4), making it a massive public health concern.

SZ is associated with metabolic complications including high insulin and glucose, insulin-resistance, metabolic syndrome, and diabetes (5, 6). SZ is also associated with an inflammatory state (7–11), which may drive SZ symptoms (9, 10, 12). Second-generation antipsychotics may exacerbate metabolic and inflammatory complications, with clozapine and olanzapine particularly implicated in insulin-resistance, poor glucose control, weight gain, type 2 diabetes mellitus (13), and increased C-reactive protein (CRP) and inflammatory cytokines (14, 15). Further, antipsychotics have serious adverse effects including extrapyramidal side effects (EPS) (e.g., pseudoparkinsonism) and sudden cardiac death, which reduce life quality and lifespan (13). Antipsychotics are also ineffective for negative symptoms, a domain which has no FDA-approved treatment (16). Therefore, alternative treatments, and ones that can potentially counter adverse effects of antipsychotic medications, are needed.

Dietary interventions have improved SZ symptoms. Our group found improvement in negative symptoms after a gluten-free diet in a sub-group of individuals with SZ with high IgG antibodies to gliadin (17). Several open label studies and case reports have shown robust benefits of a ketogenic diet (KD) for reducing metabolic complications and improving psychiatric symptoms in many psychiatric conditions (18–29), including SZ (30–34). A ketogenic diet as a medical treatment may, in part, work through bypassing glucose metabolism, impacting mitochondrial biogenesis, reducing systemic inflammation, and increasing gamma-aminobutyric acid (GABA) concentrations (35, 36). KD may also work through other biological processes relevant to neuropsychiatric illness, including decreasing oxidative stress (37), altering the gut microbiome (38), and decreasing glutamate excitotoxicity (39). We describe the case of a woman with treatment-resistant SZ (TRS) and metabolic syndrome on KD for 5 weeks and report metabolic, inflammatory, neurologic, and psychiatric outcomes in this patient.

## Case report

The participant was a 48-year-old Black woman treated with KD for 5-weeks in an inpatient setting. She passed capacity assessment for consent and consented to publishing her individual case. The Institutional Review Board at the University of Maryland, data safety monitoring boards, and the hospital research committee oversaw this study.

The patient had a DSM-5 diagnosis of SZ confirmed using the Structured Clinical Interview (SCID) (40) and maintained an antipsychotic regimen without dose change for ≥14 days. She also met criteria for KD-implementation, including body mass index >18.5, not currently pregnant or lactating, and no insulin-dependent diabetes, eating disorders, heart failure, QTc prolongation ≥ 500 ms, significant kidney or liver disease, porphyria, genetic disorders that affect fat metabolism (e.g., Gaucher disease), carnitine deficiency, pyruvate kinase deficiency, gastroparesis, or preclusions from adhering to the intervention diet (e.g., refusal or prohibitive dietary allergies/restrictions).

The participant began struggling with mental illness at age 18 and first experienced psychotic symptoms at age 21. During the 27 years since her first psychotic episode, her symptoms were unresponsive to several antipsychotic medications including haloperidol, risperidone, and aripiprazole, and only partially responsive to olanzapine before adding clozapine for her TRS. She had numerous prior hospitalizations and had been an inpatient for over 2 years prior to participation in this study. Additionally, she suffered from long-term unresponsive negative symptoms. She had a history of medication-induced type 2 diabetes and a metabolic syndrome diagnosis at KD initiation. She was also taking divalproex sodium (mood stabilization) (21 months), metformin (metabolic syndrome) (6 months), amlodipine (hypertension) (5 months), oxybutynin (enuresis) (3 months), propranolol (akathisia) (3 months), and a multivitamin, which was started alongside KD. *Pro re nata* (PRN) medications included lorazepam (akathisia, agitation, anxiety, insomnia), milk of magnesia (constipation), and acetaminophen (menstrual cramps). About 6 months prior to the study, a slow and cautious clozapine trial was initiated, given her limited response to olanzapine, which she had been on for 20 months before study initiation. When beginning clozapine, olanzapine was unable to be successfully tapered, despite slow and deliberate attempts, without worsening mood, disorganization, and psychosis. Thus, both clozapine and olanzapine were maintained. No antipsychotic changes occurred during the 5 weeks on KD to prevent introducing medication confounds. The only medication changes noted during the study were the addition of ipratropium bromide (sialorrhea) at the end of week 2, and ferrous sulphate (anemia) at week 5. Both were continued through the end of the study.

The patient was monitored by hospital staff for meal consumption on the in-patient unit and was reported to be adherent to KD, consuming only prescribed food, and finishing all or most of every meal (one-day menu in Appendix A). The participant was motivated to adhere to the diet, tolerated it well, and reported, “It’s something I have gotten used to and feel it made a positive change.”

**Figure 1:**
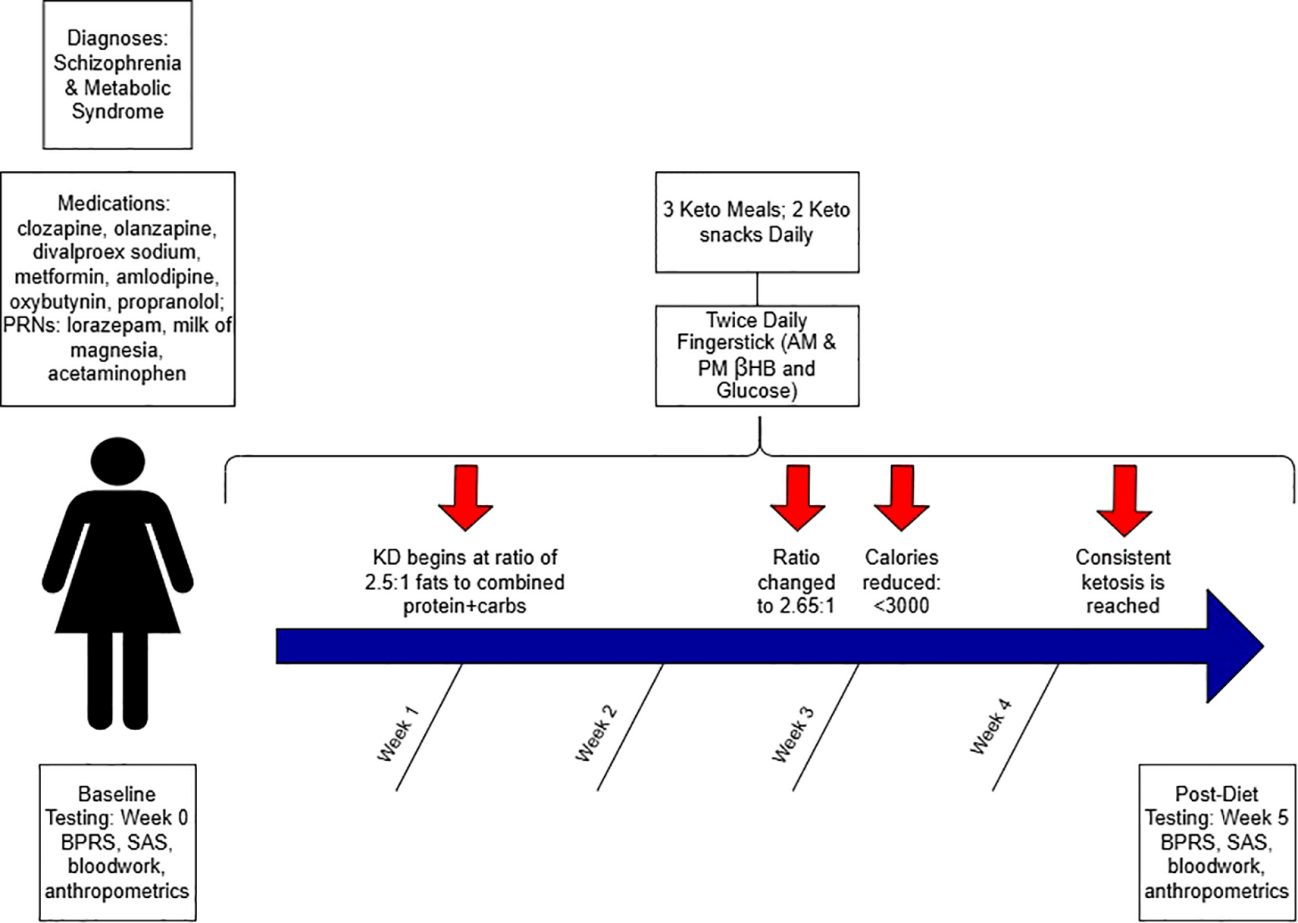
Schematic study diagram.

**Figure 2:**
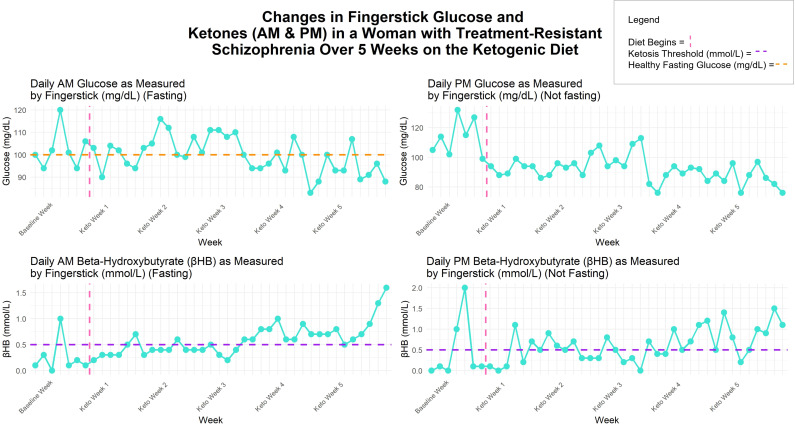
Changes in fingerstick βHB (mmol/L) and glucose (mg/dL) over 5 weeks on the ketogenic diet.

Meals were planned by the hospital dietary department and were delivered to her room. The diet ratio began as 2.5:1 fat to combined protein and carbs in grams, ~2500–3800 calories, with a macronutrient breakdown of ~70% fat, ~20% protein, ≤10% carbohydrate. Twice daily β-hydroxybutyrate (βHB) and glucose levels were obtained using a KetoMojo^®^ fingerstick (AM: fasting, PM: non-fasting). Following failure to reach consistent ketosis (βHB ≥0.5 mmol/L), her KD ratio was increased to 2.65:1 late in week 2 (macronutrient breakdown: ~73% fat, ~ 21% protein, ~7% carbohydrate), and a caloric reduction (daily <3000) occurred early in week 3 (See **Figure 1**). This improved ketosis consistency, but it was not fully met until the last 5 days (See **Figures 1**, **2**). Overall, there was an upward trend in βHB, and glucose levels decreased over time (See **Figure 2**).

Laboratory results are reported in **Table 1** where we report changes ≥20% as clinically meaningful (41). Insulin-resistance, measured by HOMA-IR, decreased by 69%, improving from prediabetic to optimal levels after 5 weeks (42). Additional metabolic markers improved, including C-peptide, which decreased by 41% as well as circulating fasting insulin, which decreased by 64%. Inflammatory markers also reduced, including IL-6, which reduced by 20%, and CRP, which reduced by 61%, the latter suggesting movement from high to average cardiac risk (43). Interestingly, these changes occurred independent of changes in weight or BMI.

**Table 1:** Pre- and post-diet outcomes in a woman with treatment-resistant schizophrenia and metabolic syndrome after a ketogenic diet for 5-weeks.

	Pre-Diet	Post-Diet	% Change
Metabolic			
HOMA-IR	1.83	0.56	-69.4%
C-peptide (ng/mL)	1.7	1	-41.2%
Fasting Insulin (µIU/mL)	7.8	2.8	-64.1%
Inflammatory			
CRP (mg/L)	7.25	2.86	-60.6%
IL-6 (pg/mL)	18.31	14.54	-20.1%
Anthropometric			
Weight (lbs)	210.6	208.8	-0.9%
BMI	36.7	35.4	-3.5%
Clinical Assessments			
SAS	15	3	-80.0%
BPRS	43	48	+11.6%
Sub-domains:			
Negative	10	8	
Positive	6	7	

Antipsychotic-related EPS (i.e., pseudoparkinsonism), measured by the Simpson-Angus Scale (SAS) (44), reduced dramatically (15 to 3; -80%). Baseline score indicated a severe movement disorder, while post-diet level reflected a mild movement disorder, bordering on none. The Brief Psychiatric Rating Scale (BPRS) (45) total score was used to measure global psychopathology with symptom domain subscales calculated and included in **Table 1**. We noted a decrease in negative symptoms; however, her positive symptoms were rated higher at week 5 (physician and study team noted unit stressors potentially confounding the endpoint rating; more on this in the discussion), resulting in no dramatic change in total BPRS score (See **Table 1**). Clinical assessments were conducted by a blinded rater who was unaware of the participant's dietary intervention. Moderate side effects of tinnitus, hypersalivation, and stiffness were unlikely to be related to the diet but improved on KD. Keto flu symptoms were absent most weeks and were never rated above mild.

## Discussion

This case describes metabolic, inflammatory, neurologic, and psychiatric outcomes in a woman with TRS and metabolic syndrome after 5 weeks on KD in an inpatient setting. Meals were curated by hospital dieticians, and the rater for clinical assessments was blinded to the participant's dietary intervention, mitigating bias.

This patient experienced improvement in metabolic markers of HOMA-IR, fasting insulin and C-peptide, and a downward trend throughout the diet in twice daily fingerstick glucose levels. This is notable given her history of type 2 diabetes and metabolic syndrome upon KD initiation and that she was only in ketosis consistently for 5 days. Additionally, inflammatory markers, CRP and IL-6, notably reduced, with the former bringing her risk for a cardiac event from high to average. IL-6 is implicated in SZ pathophysiology (46), and its reduction relates to successful SZ treatment (47). These are robust improvements despite a short time on diet therapy, inconsistent ketosis, and without a notable change in weight/BMI. Reducing several cardiac and metabolic risk factors could improve metabolic disease course and life expectancy in people taking antipsychotics.

Notably, EPS dramatically improved, aligning with reports on KD and Parkinson’s disease (48). If this holds in larger studies, KD could be extremely impactful for countering such a debilitating adverse effect of antipsychotic medications. It is possible that limited gluten consumption on KD contributed to improvements in neurological side effects, as there have been cases of neurologic improvements after a gluten-free diet in individuals with SZ (49). Neurologic issues such as gluten ataxia are seen in people with antibodies to gliadin, which is a protein within gluten, and the SZ population has high prevalence of these antibodies (50, 51). Also, KD is highly effective for epilepsy (52) and has shown efficacy in other neurologic diseases as well (53). Results from a Parkinsonian mouse model suggested that βHB, through its downstream conversion to succinate in the citric acid cycle, is neuroprotective through its ability to increase the rate of oxygen consumption through mitochondrial protein complex II and can improve parkinsonian-like motor deficits (54).

Given the participant's very short time consistently in ketosis (5 days), we did not expect to see significant improvement in her total BPRS score. While some improvements have been noted as early as two weeks (30, 34), other studies report maximum benefits over longer periods of time (31, 32). We did see a hint for decrease in negative symptoms, which is a notable improvement in this patient, who had not previously experienced relief in this domain, as is typical in TRS (55). Her week 5 positive symptom ratings were higher than baseline, which her psychiatrist and research staff believed was due to a series of upsetting altercations with another patient on the unit during the last two weeks of the study and menstruation during the last week, which is associated with worse psychosis in women (56, 57).

Despite endpoint stressors, our patient verbally reported many times that she benefitted from KD and stated she would like to try it again. From the reported data, it is noted that inconsistent ketosis, the short dietary duration, and the week 5 stressors precluded us from seeing global or positive symptom improvement that has been reported previously after KD in individuals with SZ (30–34). We acknowledge that these factors limit the generalizability of our case and comparability to other KD case studies and clinical trials. However, previous trials reported imperfect ketosis in their samples as well (32, 33), though to a lesser extent than our case. Ongoing clinical trials for KD in psychiatry are considering baseline ratings once the patient reaches ketosis, a design that mitigates intervention time spent out of ketosis and titration of diet during the intervention period, notable limitations of our study.

The delay in reaching βHB ≥ 0.5 mmol/L may be due to clozapine and olanzapine, which are known to induce insulin resistance, but also may be due to a high caloric diet at the beginning (some days > 3000 calories) (See **Figure 2**). However, even with inconsistent ketosis, our patient experienced large improvements in cardiometabolic and inflammatory markers and antipsychotic-associated Parkinsonian-like symptoms. This is intriguing as our data suggest that consistently meeting the ketosis threshold of βHB ≥ 0.5 mmol/L may not be necessary for certain metabolic, inflammatory, and neurological improvements, and that significant health benefits can occur on KD in a short amount of time. However, emerging clinical studies on KD for psychiatric symptom improvement suggest an optimal βHB range between 1–3 mmol/L (20). It remains unknown if the observed benefits, or additional ones, could occur in individuals with SZ in response to other low carbohydrate diets, such as the Mediterranean diet, which has been shown to improve depression (58) and relevant metabolic health markers (59) in samples without SZ.

Our case shines light on other needed data in the field. The potential that KD could improve negative symptoms would be revolutionary as current treatments for negative symptoms are lacking. Also, testing the use of other ketosis-boosting strategies, such as exogenous ketones for SZ treatment, may be an alternative and has shown benefits in other psychiatric and neurologic conditions (60). Lastly, as we advance in precision medicine psychiatry (61), determining genetic variants or biomarkers associated with treatment response will help guide us in selecting which patients may best respond to KD. In the epilepsy field, genetic variants may help predict which patients will have the most robust treatment response to KD (61–64). Thus, a genetic-metabolic approach may help guide future treatments in nutritional psychiatry.

This is the first case to report therapeutic effects of KD on inflammatory markers and antipsychotic-associated EPS in a person with SZ, and the first to report effects of KD specifically in someone with TRS. We also hint at the possibility that negative symptom improvements may be seen even in people with TRS. These results may suggest KD as a potential adjunct treatment to antipsychotics to counter adverse effects, improve quality of life, and life expectancy, if positive data continues to accumulate. This case, as well as other studies (30–34), demonstrate diverse health benefits of KD for individuals with mental illness, suggesting the need for continued research in this area (65). We also point out the challenges in this study of reaching ketosis in a TRS patient on metabolically impactful antipsychotics and that even low levels of ketosis could vastly improve metabolic health. Well-controlled clinical trials with patients in therapeutic ketosis are needed to establish the effects of KD as a treatment for SZ and, more specifically, TRS, and on related biomarkers and health factors.

## Data Availability

The original contributions presented in the study are included in the article. Further inquiries can be directed to the corresponding authors.

## Appendix A

**Table:** Example ketogenic diet menu (1 Day*; 2855 calories)*.

Meal	Food items
Breakfast	Sausage & eggs
Lunch	Seasoned ground beef; green peppers; salad
2 pm Snack	Pecans & cashews
Dinner	Chicken with green beans; macadamia nuts; salad
8 pm Snack	KetoCal^®^ 4:1 formula - vanilla/chocolate
